# The Annealing Helicase and Branch Migration Activities of *Drosophila* HARP

**DOI:** 10.1371/journal.pone.0098173

**Published:** 2014-05-27

**Authors:** George A. Kassavetis, James T. Kadonaga

**Affiliations:** Section of Molecular Biology, University of California San Diego, La Jolla, California, United States of America; St. Georges University of London, United Kingdom

## Abstract

HARP (SMARCAL1, MARCAL1) is an annealing helicase that functions in the repair and restart of damaged DNA replication forks through its DNA branch migration and replication fork regression activities. HARP is conserved among metazoans. HARP from invertebrates differs by the absence of one of the two HARP-specific domain repeats found in vertebrates. The annealing helicase and branch migration activity of invertebrate HARP has not been documented. We found that HARP from *Drosophila melanogaster* retains the annealing helicase activity of human HARP, the ability to disrupt D-loops and to branch migrate Holliday junctions, but fails to regress model DNA replication fork structures. A comparison of human and *Drosophila* HARP on additional substrates revealed that both HARPs are competent in branch migrating a bidirectional replication bubble composed of either DNA:DNA or RNA:DNA hybrid. Human, but not *Drosophila*, HARP is also capable of regressing a replication fork structure containing a highly stable poly rG:dC hybrid. Persistent RNA:DNA hybrids *in vivo* can lead to replication fork arrest and genome instability. The ability of HARP to strand transfer hybrids may signify a hybrid removal function for this enzyme, *in vivo*.

## Introduction

HARP (*h*ep*A*-*r*elated *p*rotein; also called SMARCAL1 in *Homo sapiens* (*hs*) and MARCAL1 in *Drosophila melanogaster* (*dm*)) is a distant member of the SNF2 family of helicase-like ATPases. Biallelic mutations in *hs*HARP cause the multisystem disorder, Schimke immuno-ossious dysplasia (SIOD) [1]. Members of the SNF2 family ATPases have diverse functions, such as, chromatin remodeling, DNA repair, replication, recombination, and transcription [2]. HARP is categorized as an ATP-dependent annealing helicase based on its ability to rewind complimentary single stranded (ss) DNA that is otherwise stably maintained by the ssDNA-binding protein, Replication Protein A (RPA) [3]. *hs*HARP does not bind stably to ssDNA or fully double stranded (ds) DNA, but does bind with high affinity to a number of DNA structures, including DNA forks, ssDNA:dsDNA junctions with extended 5′- or 3′-ssDNA overhangs, heteroduplex DNA bubbles, internal ssDNA gaps and Holliday junctions. These structures likewise optimally stimulate the DNA-dependent ATPase activity of *hs*HARP [4], [3].

**Figure 1:**
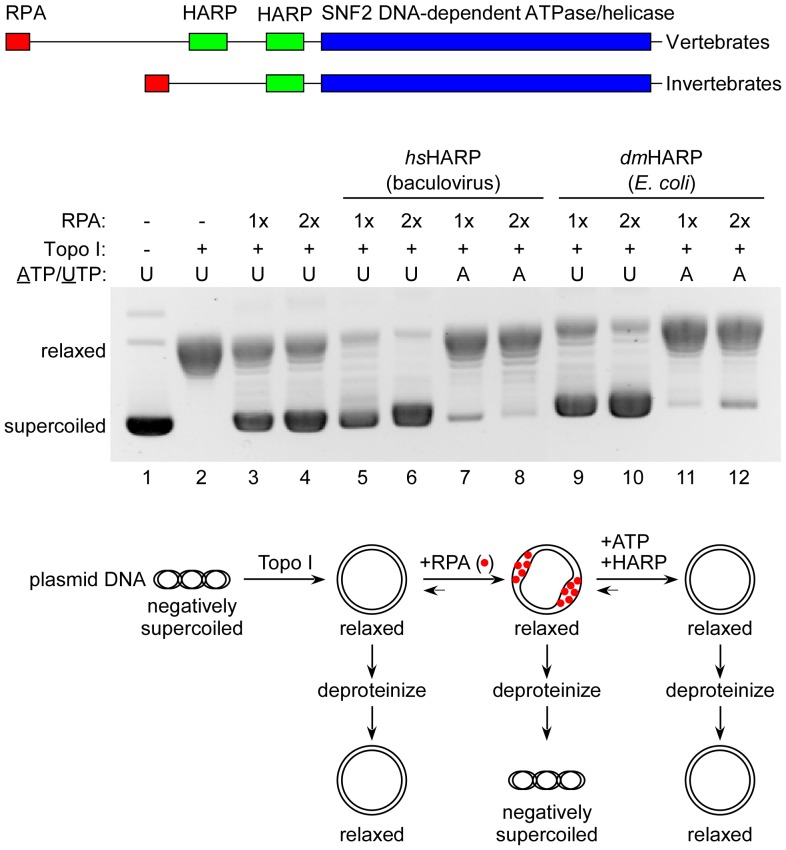
*dm*HARP is an ATP-dependent annealing helicase. Top: homology domains of vertebrate and invertebrate HARP. Middle: The presence of *hs*HARP from baculovirus-infected insect cells or *dm*HARP expressed in *E. coli*, topoisomerase I, RPA, and UTP or ATP in each analyzed reaction are specified above the gel image. The concentrations of *hs*HARP and *dm*HARP were 300 and 150 nM, respectively. 1x RPA is 800 nM. Relaxed and supercoiled plasmids are identified at the left. Topoisomerase I generates relaxed plasmids (lane 2). The ssDNA binding of RPA generates supercoiled plasmids in the presence of topoisomerase I (*e.g.*, lane 3). The ATP-dependent annealing helicase activity of HARP releases RPA, generating a relaxed plasmid in the presence of topoisomerase I (*e.g.*, lane 7). Bottom: diagrammatic description of the annealing helicase assay.

HARP contains an RPA binding motif near its N-terminus and either one (invertebrates) or two adjacent (vertebrates) HARP-specific domains at the N-terminal border of its SNF2 ATPase domain (Figure 1, top). The location of the invertebrate HARP domain is equivalent to the second HARP domain repeat found in vertebrates. The RPA binding motif of HARP is not essential for its annealing helicase activity *in vitro* [5], [6]. Deletions and point mutations have also shown that the N-proximal HARP domain of *hs*HARP is also not required for annealing helicase activity and that a minimal region from the second HARP domain to the C-terminal end of the SNF2 ATPase domain suffices [4]. Indeed, fusing the two HARP domains to the N-terminus of other SNF2 family proteins confers annealing helicase activity [6].

HARP is a DNA replication stress response protein that is recruited to sites of DNA damage or stalled/arrested replication forks through its interactions with RPA which accumulates at the resultant ssDNA gaps present at these sites [5], [7], [8], [9]. HARP also appears to be associated with unperturbed replication forks [4]. Although *hs*HARP does not contain a strict (unwinding only) ATP-dependent helicase activity, it does contain a robust branch migration (concomitant unwinding and annealing) activity capable of disrupting plasmid-borne D-loops, resolving Holliday junctions, regressing replication fork structures and restoring replication forks from its regressed (chicken-foot) state ([10], [4], [11].

HARP functions in the repair of damaged replication forks and facilitates the restart of arrested replication forks through its branch migration activity. A recent and enlightening study [11] indicates that RPA enforces a substrate preference for HARP regression activity at replication forks containing leading strand gaps (that would result from leading strand damage) and enforces a substrate preference for HARP-mediated restoration of the replication fork when the chicken foot regression product contains a longer 3′-tail (that would restore a normal replication fork with lagging strand gaps).

Despite the clear role of HARP in DNA repair, replication fork restart and the presence of the SIOD disease phenotype of biallelic *hs*HARP mutations, a biallelic deletion in mouse HARP, that removes both the RPA binding motif and the first HARP domain repeat and a biallelic deletion in *dm*HARP that results in non-expression, did not display significant growth defects in mice and flies under non-stressful environmental conditions [12]. In mice, it is conceivable that there may be functional redundancy between HARP and its annealing helicase paralog, Annealing Helicase 2 (AH2; also termed ZRANB3) [13], [10], [14], but no other annealing helicase has been identified in *Drosophila*. Conceivably, there is another invertebrate annealing helicase that has yet to be discerned by sequence homology to either HARP or AH2. Alternatively, the type of events that lead to replication fork arrest in vertebrates that are particularly suited to HARP action, are rare in *Drosophila* under non-stressful growth conditions [12].

In this manuscript, we examined the ability of *dm*HARP, which naturally contains only one HARP domain, to branch migrate DNA structures that were previously shown to serve as substrates for *hs*HARP. In addition, we examined the ability of *hs*HARP and *dm*HARP to branch migrate bidirectional replication bubbles, replication bubbles that contain an RNA:DNA hybrid, and a replication fork containing a highly stable poly rG:dC hybrid. RNA-containing structures are of interest, since R-loops, and in particular stabile rG-rich hybrids, which may result in G-quadruplex structures in the opposing non-transcribed DNA strand, can persist following transcription *in vivo* and *in vitro*, and have been implicated in genome instability, replication fork and transcriptional elongation arrest (for reviews, see [15], [16], [17]). We found *hs*HARP and *dm*HARP displayed comparable annealing helicase, D-loop disruption, branch migration of Holliday junctions, and branch migration of DNA and RNA:DNA hybrid-containing bidirectional replication bubbles, activities. Importantly, *hs*HARP was capable of regressing replication forks containing a highly stable poly rG:dC hybrid. In contrast, *dm*HARP was unable to regress standard DNA replication forks structures and replication forks containing the poly rG:dC hybrid.

## Results and Discussion

We have purified *Drosophila* (*dm*) HARP based, in part, on its reported potential (*c.f.*, [12], [18]) to affect steps during the RNA polymerase II transcription cycle (initiation, elongation and termination). Both a bacterial and a baculovirus-infected insect cell expression system were employed as the source for purification as the former is more amenable for generating modified proteins and for structural studies. The catalytic properties of a bacterially-expressed HARP has not been reported, nor have the potential differences between human (*hs*) HARP, with its two HARP domains, and *dm*HARP, with its one HARP domain, been examined (Figure 1, top). The purity of the *E. coli*-expressed *dm*HARP preparation was comparable to that of *dm*HARP and *hs*HARP derived from insect cells (Figure S1).

*dm*HARP, expressed in *E. coli*, manifested an annealing helicase activity (Figure 1). In this assay (diagrammed below the gel image), the ssDNA binding protein RPA binds to a partially unwound supercoiled plasmid. In the presence of topoisomerase I and bound RPA, the plasmid remained supercoiled upon deproteinization (lanes 3 and 4), but in the absence of bound RPA, the plasmid became relaxed (lane 2). In the presence of a hydrolysable NTP (ATP), *hs*HARP reannealed the DNA strands, releasing RPA, such that the plasmid again became relaxed (lanes 7 and 8), but not when a non-hydrolysable NTP (UTP) was used (lanes 5 and 6). The annealing helicase activity of *E. coli*-expressed *dm*HARP was comparable (compare lanes 11 and 12 with lanes 9 and 10). In the absence of ATP, both HARPs increased the superhelical density of the plasmid (compare lanes 5, 6, 9 and 10 with lanes 3 and 4). This effect is likely due to high affinity binding of HARP to ds:ss DNA fork junctions [4], [3]. The HARP-mediated stabilization of ds:ss fork junctions should increase the amount of RPA:ssDNA complexes on plasmid DNA and both bound HARP and RPA should increase the superhelical density in the presence of topoisomerase I upon subsequent deproteinization. *dm*HARP purified from insect cells was less active in this is assay (Figure S2).

**Figure 2:**
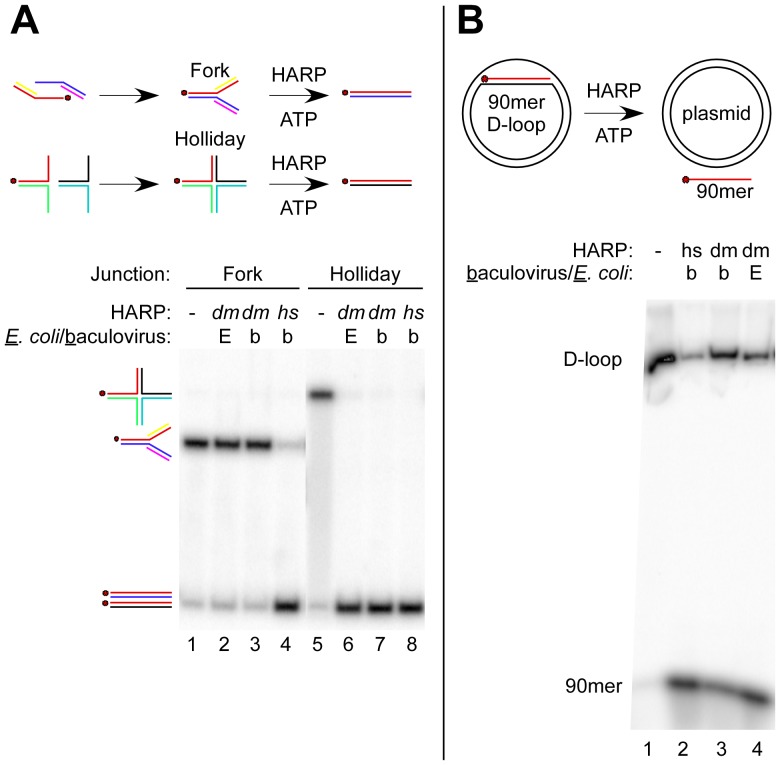
The branch migration activities of *dm*HARP and *hs*HARP differ. (**A**) *dm*HARP is competent for catalyzing Holliday junction migration but not replication fork regression. The reaction scheme is shown above the figure along with whether *dm*HARP was expressed in *E. coli* [E] or baculovirus-infected insect cells [b]. The gel migration of the replication fork and Holliday junction substrates is shown at the left along with the dsDNA products. The red dot signifies the common 5′-^32^P-labeled oligo (A60; Material and Methods). Lanes dealing with an unrelated helicase were removed between lanes 4 and 5. (**B**) *dm*HARP is competent in disrupting D-loops. D-loops were formed with a supercoiled plasmid and a labeled 90mer DNA oligonucleotide, chromatographically purified and incubated with *dm*HARP or *hs*HARP expressed in *E. coli* or baculovirus-infected cells as indicated above the gel image. The D-loop and 90mer are identified at the left.

*hs*HARP has been shown to contain a robust, ATP-dependent branch migration activity at Holliday junctions and replication forks [4], [11], [10]. Holliday and replication fork junctions were formed by annealing the individual halves (Figure 2A, top) with single bp mismatches to prevent spontaneous branch migration [4]. Both *hs*HARP and *dm*HARP (from *E. coli* and insect cells) catalyzed the branch migration of Holliday junctions to generate a dsDNA product (compare lanes 6–8 to lane 5). Surprisingly, unlike *hs*HARP (lane 4), *dm*HARP from either source was unable to regress a 4-strand DNA replication fork junction (compare lanes 2 and 3 to lane 1). We also commonly observed that when the two halves of the Holliday and replication fork junctions were not pre-annealed, *hs*HARP and *dm*HARP increased the annealing rate of the two halves during the final incubation (Figure S3). To test whether *dm*HARP was competent to disrupt D-loops as previously shown for *hs*HARP [10], a 90 bp D-loop was formed on a supercoiled plasmid with RecA and RPA and subsequently purified (Figure 2B, lane 1). Both *hs*HARP and *dm*HARP catalyzed D-loop disruption (lanes 2-4), but the reactions with *dm*HARP were less complete within the time frame of this assay.

**Figure 3:**
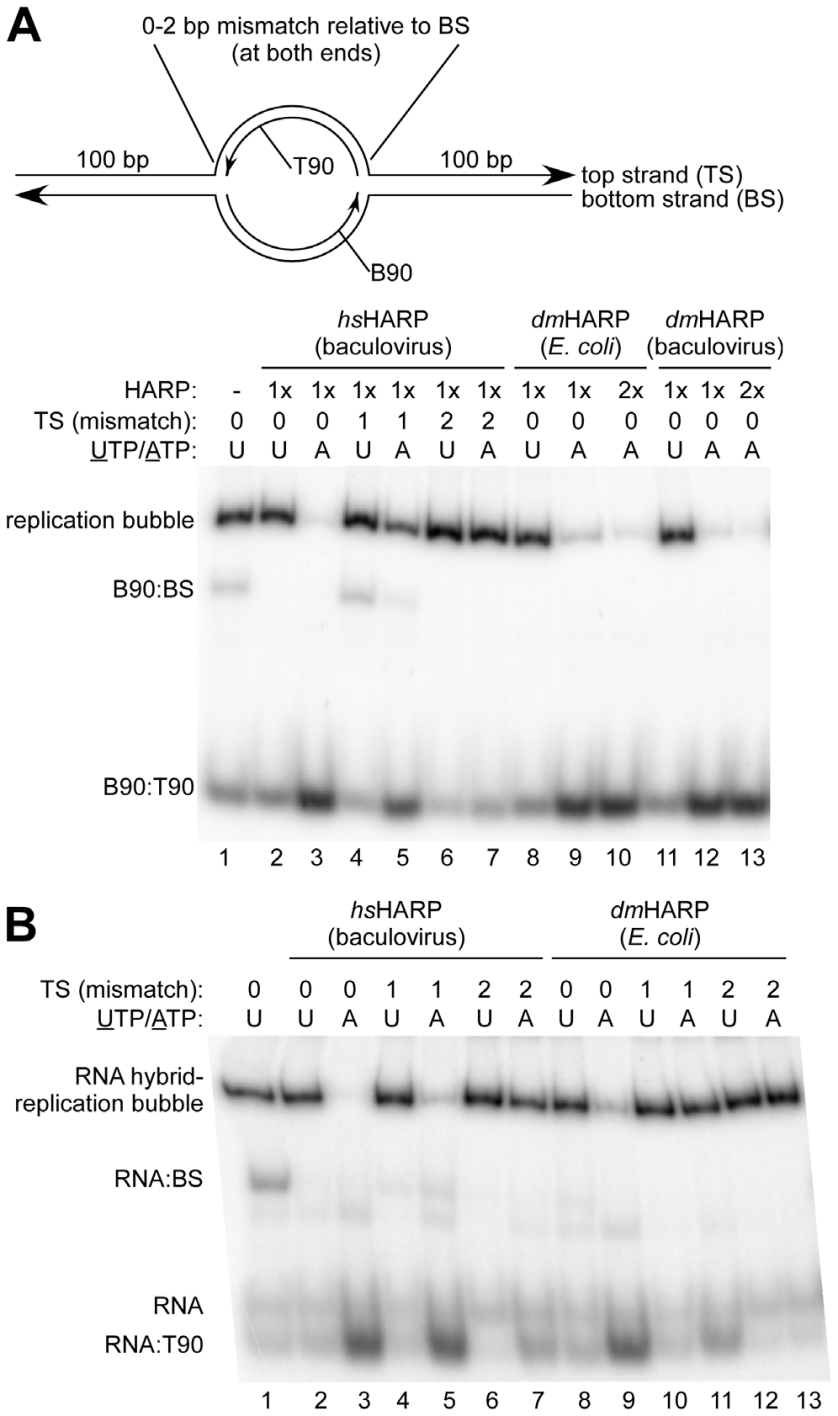
HARP disassembles bidirectional replication bubbles containing an RNA:DNA hybrid. (**A**) *hs*HARP and *dm*HARP can disassemble a bidirectional replication bubble. Top: A 90 bp, centrally located, replication bubble was formed with oligonucleotides B90 (5′-^32^P-labeled) and T90 pre-annealed to 290 nt bottom (BS) and top (TS) strands, respectively. Variants of TS with 1 or 2 bp mismatches relative to the BS at both ends of the 90 bp bubble were also used. Bottom: The presence and source of HARP (1x is 20 nM), bp mismatches in the TS, and the presence of UTP or ATP are indicated above the gel image. The replication bubble, non-annealed B90:BS and the B90:T90 branch migration products are identified at the left. (**B**) *hs*HARP and *dm*HARP (at 20 nM) can disassemble a replication bubble containing an RNA:DNA hybrid. Annotation follows (**A**).

Since *dm*HARP was unable to catalyze regression of a model replication fork, we explored other replication fork-containing structures that were less prone to spontaneous fork migration in the absence of stabilizing 1 bp mismatches. To this end, the branch migration activity of HARP was extended a bidirectional replication bubble (Figure 3A, top), mimicking an origin of replication. Replication bubbles halves were formed by annealing centrally located 90mers (T90 and 5′-^32^P-labeled B90) to 290 nt top (TS) and bottom (BS) strands (Table S1), respectively, and subsequently annealed together. Variants of the top strand were also used with 1 or 2 bp mismatches relative to each end of the T90 and to the bottom strand. Both *hs*HARP and *dm*HARP (from *E. coli* and insect cells) branch migrated non-mismatched bubbles to form a labeled B90:T90 duplex (ATP-containing lanes 3,9 and 12 compared to UTP-containing lanes 2, 8 and 11, respectively). A 2 bp mismatch in the top strand at each end of the replication bubble effectively prevented the branch migration activity of *hs*HARP (lanes 6 and 7). In the absence of annealed T90 (to form a D-loop), the 2 bp mismatches did not prevent spontaneous bubble collapse (not shown). A 1 bp mismatch in the top strand at each end of the bubble substantially reduced the conversion of the replication bubbles to duplex DNA by *hs*HARP from 74±4% (SD, n = 3) (lane 3 *versus* 2, and data not shown) to 26±7% (SD, n = 3) (lane 5 *versus* 4, and data not shown). This effect is curious since the single replication fork junctions used in Figure 2A, Figure S3 and an additional experiment also contain a 1 bp mismatch at the same location, and 72±7% (SD, n = 3) of these forks were regressed by *hs*HARP. This difference between the replication bubble and fork was not due to the additive effect of 1 bp mismatches at each end of the bubble since an intransigent 5 bp mismatch at *either* end of the replication bubble had little or no effect on the ability of *hs*HARP to disrupt these bubbles (Figure S4).

Since transcription continues during S phase and persistent RNA:DNA hybrids can potentially lead to replication fork arrest, a replication bubble containing and RNA:DNA hybrid was generated by replacing the labeled B90 with a labeled 109 nt RNA transcript. This RNA extends base pairing 15 and 4 bp to the left and right, respectively, to that of B90 at the top of Figure 3A. Both *hs*HARP and *dm*HARP were competent in branch migrating the RNA:DNA hybrid (Figure 3B, compare lanes 3 to 2 and 9 to 8, respectively). In contrast to the DNA replication bubble in Figure 3A, *hs*HARP efficiently removed the hybrid when 1 bp top strand mismatches were present at both ends of the T90-defined bubble (Figure 3B, lanes 5 and 4) and *hs*HARP was weakly active when 2 bp top strand mismatches were present at both ends of the T90-defined bubble (lanes 7 and 6). *dm*HARP likewise displayed a slight, but detectable, branch migration activity with the 1 bp mismatch substrate (lanes 10 and 11). We suggest that the greater ease of branch migrating these hybrid replication forks may be a consequence of the mismatch not being confronted at the initial step of branch migration as was the case in Figure 3A. However, the sequence context of a mismatch, when presented at the initiation of branch migration, must also matter since the *hs*HARP branch migration efficiency with the 1 bp mismatch present in the replication fork used in Figure 2A and the 1 bp mismatches in the replication bubble used in Figure 3A differ. In regard to assessing potential *in vivo* functions of *dm*HARP, the capability of *dm*HARP to regress a DNA replication fork was only clearly apparent in the context of a fully complementary bidirectional replication bubble.

**Figure 4:**
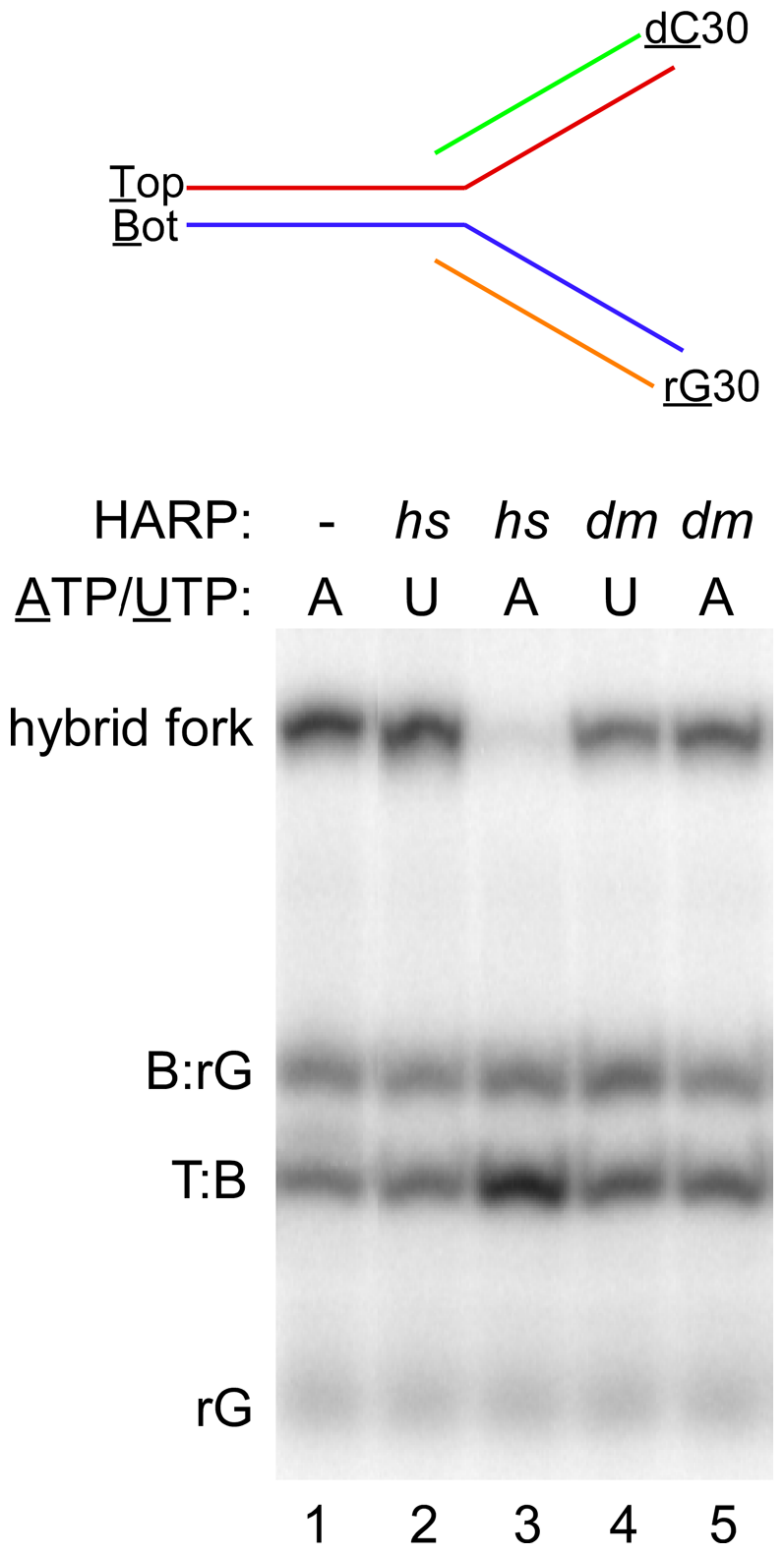
*hs*HARP regresses replication forks containing a stable poly rG:dC hybrid. 5′- labeled poly rG30 and unlabeled poly dC30 were annealed to 5′-labeled 60mers Bot and Top, respectively, and subsequently annealed to each other (diagramed at the top). The presence of 40 nM *hs*HARP and *dm*HARP (from *E. coli*) and the presence of ATP or UTP are indicated above each lane. The gel migration of the hybrid fork, the 60 bp T:B duplex regression product, excess B:rG30 and rG30 starting materials are indicated at the left of the gel. Replicates of the reactions analyzed in lanes 2–5 were excised between lanes 1 and 2.

As noted in the Introduction, RNA:DNA hybrids containing poly rG stretches are extremely stable, can persist or reform following transcription *in viv*o, can generate G quadruplexes with the nontemplate strand and appear to cause genome instability, replication fork and transcription elongation arrest. Since the melting temperature of poly rG:dC hybrids are significantly higher than its poly dG:dC counterpart (*e.g*., by >20°C at 100 mM NaCl; [19]), fully complementary 3-strand and 4-strand fork junctions could be formed with a 30 bp poly rG:dC hybrid at the fork end without the complication of spontaneous strand displacement via fork migration. *hs*HARP was competent in catalyzing the regression of a 4-strand fork containing the poly rG:dC hybrid (Figure 4, compare lanes 2 and 3) whereas *dm*HARP was inactive with this fully complementary substrate (lanes 4 and 5). Both *hs*HARP and *dm*HARP were not able to displace this RNA from a 3-strand fork junction (Figure S5). Given that, *hs*HARP was capable of disrupting a D-loop (Figure 2B) and the rG30 hybrid in the 4-strand junction (Figure 4), but not the 3-strand junction that is equivalent to the D-loop suggests that in addition to ATP hydrolysis, concomitant annealing of rG30 with dC30 is necessary component for overcoming the energy barrier imposed by this stable hybrid.

The ability of HARP to branch migrate an RNA:DNA hybrid may have functional significance. In addition to digestion by RNase H's, a number of helicases have been shown to act on RNA:DNA hybrids, including WRN [20], BLM [21], PIF1 [22] and the MCM complex replication helicase [23]. HARP may participate in the removal of R-loops that stall replication fork progression through its capacity of transferring the RNA from an already synthesized lagging or leading strand. The co-binding of HARP and the 3′-exonuclease and 3′-helicase WRN to different subunits of RPA at the replication fork [24] suggests that these enzymes could potentially partner in RNA:DNA hybrid removal during replication.

## Materials and Methods

### Proteins

The coding sequence of *dm*HARP from the *Drosophila* Genomics Resource Center cDNA plasmid RE44811 was cloned as an N-FLAG-, C-His_6_-tagged protein into the *E. coli* expression vector pET21d and into the baculovirus expression vector pFastbac1 (with the amino acid sequence DYKDDDDK inserted following the N-terminal M and VEHHHHHH inserted at the C-terminus). *dm*HARP was expressed in Rosetta pLysS (Novagen) overnight at 15°C in a modified LB medium containing 0.2% (w/v) NaCl, 0.7 M sorbitol, 2.5 mM betaine with 100 µM IPTG. All purification steps were maintained at 0–4°C. The cell pellet (6 g) was resuspended in 7 volumes of buffer L (40 mM Tris-Cl, pH 8.0, 0.01% (v/v) Tween 20, 10% (v/v) glycerol, 10 mM 2-mercaptoethanol, 1 µg/ml pepstatin, 1 µg/ml leupeptin and 0.5 mM phenylmethylsulfonyl fluoride) containing 535 mM NaCl, 300 µg/ml lysozyme, 1 mM Na_2_S_2_O_5_, 1 mM benzamidine and 1 µg/ml aprotinin. Following 10 rounds of sonication, the lysate was clarified by centrifugation (1 h at 38,000×g), adjusted to contain 10 mM imidazole, and loaded onto a 1 ml NiNTA Sepharose column equilibrated in buffer H+500 mM NaCl (buffer L with 30 mM NaHEPES, pH 7.8, in place of Tris-Cl and including 10 mM imidazole, and 1 µg/ml aprotinin). The column was washed in the same buffer (10 ml), buffer H+500 mM NaCl with 20 mM imidazole (5 ml) and eluted in buffer H+500 mM NaCl containing 200 mM imidazole and 15% (v/v) glycerol (1 ml following a 0.5 ml pre-elution fraction). One half of this material was loaded onto a 0.5 ml anti-FLAG M2 agarose (Sigma) column equilibrated in buffer HM+500 mM NaCl (buffer H with 20 mM NaHEPES, 1.5 mM MgCl_2_ and NP40 in place of Tween 20, but lacking imidazole and aprotinin), washed with 6 ml of the same buffer, 2.5 ml buffer HM+100 mM NaCl and eluted with 1.4 ml buffer HM+100 mM NaCl with 200 µg/ml 3xFLAG peptide (Sigma). *dm*HARP was stored at -80°C or diluted into HARP storage buffer (20 mM KHEPES, pH 7.8, 100 mM KCl, 0.01% (v/v) NP40, 1 mM dithiothreitol (DTT), 200 µg/ml BSA, 50% (v/v) glycerol, 1 µg/ml pepstatin, 1 µg/ml leupeptin and 0.5 mM phenylmethylsulfonyl fluoride) and stored at −20°C without significant loss in activity. HARP diluent (storage buffer with 10% (v/v) glycerol) was used for further dilutions and in place of *dm*HARP for assays.

FLAG-tagged *dm*HARP and *hs*HARP were expressed in 1 L baculovirus-infected Sf9 culture cells and purified as described [3] except that following batch binding to 1 ml M2 agarose, the resin was washed and eluted in column format as described for *dm*HARP above with volumes increased 2-fold. Storage buffer and diluent were as specified above. RPA was purified as described [3]. The catalytic domain of *Drosophila* topoisomerase I was purified as described [25] and was kindly provided by Sharon Torigoe. The purity of these protein preparations is shown in Figure S1. All proteins were quantified relative to a BSA standard curve on Coomassie-stained gels.

### Assays

All reported assay results were replicated in separate experiments at least two times.

#### Annealing helicase assay

The annealing helicase assay was performed as described [3] with the following changes in protein and DNA components: 400 ng supercoiled pU6Rext [26], 800 nM RPA, 80 nM topoisomerase I, and 150 or 300 nM HARP.

#### Replication fork regression and Holliday junction migration assays

The oligonucleotides used were derived from [4]: A60 = oligo#40, A30 = #50, B60 = #52 without the 3′-end dCMP, B30 = #53, C60 = #54 without the 3′-end dTMP, E60 = #55, and D60 = #56 without the 5′-end dT. These sequences generate a 1 bp mismatch that prevents spontaneous strand migration upon annealing. A60, common to the replication fork and Holliday junction was 5′-^32^P-labeled. Replication fork (A60:A30 and B:60:B30) and Holliday junction (A60:C60 and D60:E60) halves were annealed and then annealed together in 20 mM Tris-Cl, pH 7.5, 5 mM MgCl_2_, 5 mM DTT, 0.5 mM EDTA, and 40 mM KCl. The reactions were performed in the same buffer containing 25 mM KCl, 2 mM ATP, 5 nM replication forks or Holliday junctions for 30 min at 30°C with 20 nM *dm*HARP or at 37°C with 20 nM *hs*HARP. The reactions were stopped with a gel loading buffer providing 7 mM EDTA and 0.3% SDS for electrophoresis on an 8% polyacrylamide gel in 1xTBE. When cited in the main text, the disruption of replication forks (F) was quantified from full lane width image density profiles following removal of phosphorimage plate background. Total image density in each lane (T) was used to correct for minor, between-lane loading variation. Percent replication fork disruption  = [(F/T^-HARP^–F/T^+HARP^)/(F/T^-HARP^)]×100.

#### D-loop disruption assay

D-loops were formed starting with a 20 µl reaction mixture containing 25 mM TrisOAc, pH 7.8, 10 mM Mg(OAc)_2_, 1 mM DTT, 2.5 mM γ-S-ATP, 100 µg/ml BSA, 10 nM 5′-^32^P-labeled 90mer oligo DL90 (Table S1) and 0.3 µM RecA (NEB) for 5 min at 37°C. Two µl of 1 µM RPA was added for an additional 5 min, followed by 1 µl of 200 nM plasmid, pU6Rext, for 5 min. The reaction was stopped by the addition of 1.2 µl of 10% (w/v) SDS and the plasmid purified by chromatography on a 0.5 ml Sepharose 2B column equilibrated in 20 mM TrisOAc, pH 7.8, 5 mM Mg(OAc)_2_, 2 mM DTT, 0.01% (v/v) NP40, 30 mM KCl and 100 µg/ml BSA. The excluded peak fraction (50 µl) was adjusted to contain 5 mM ATP and 12 µl was distributed to tubes containing 3 µl of 200 nM HARP or HARP diluent and incubated at 30°C (*dm*HARP) or 37°C (*hs*HARP) for 20 min. The reaction was terminated by the addition of 4xFicoll sample buffer containing 2.5% (w/v) SDS for electrophoresis on a 2.5% polyacrylamide–0.5% agarose gel in 1xTBE.

#### Replication bubble disruption assays

Briefly, bidirectional replication bubbles were formed with 290 nt separated DNA strands [27] and centrally-located, complementary, 90mer oligonucleotides. Oligonucleotides B90 and T90 (Table S1) were annealed and used as PCR template for introducing an *Eco*RI site at the 5′-end of B90 and a *Hind*III site at the 5′-end of T90 for insertion into vector pGEM1 (Promega). Additional *Eco*RI and *Hind*III site primers were used to introduce 1, 2 or 5 bp transversion mutations at each or either 5′-end of the B90 and T90 sequence for pGEM1 insertion. The resulting plasmids were used as PCR templates to generate the 290 nt bottom strand with primers +150 3′-ribo (indicating a ribonucleotide-3′ end) and −100 U and 290 nt top strands (including the transversion variants) with primers −150 3′-ribo and +100D (Table S1). The purified PCR products were adjusted to contain 0.1 N NaOH and incubated overnight at 37°C to cleave off the 3′-ribo primers, neutralized with HCl, ethanol precipitated, resuspended in formamide, and the 290 nt cleaved strand eluted from an 8% polyacrylamide gel containing 8 M urea following visualization by UV shadowing. 5′-^32^P-labeled B90 was annealed to the 290 nt bottom strand (BS) and T90 was annealed to the top strand (TS) or the TS transversion mutation variants. The RNA used in place of B90 for annealing to the bottom strand was generated by transcription with T7 RNA polymerase using the non-mutated pGEM1 clone cleaved at the *Hind*III site and 5′-^32^P-labeled with guanylyltransferase (NEB). Replication bubbles were formed with 5 nM B90:BS or RNA:BS and 6 nM T90:TS (or the TS variants) in 8 µl 20 mM NaHEPES, pH 7.8, 5 mM MgCl_2_, 50 mM NaCl, 2 mM DTT, 0.01% (v/v) NP40, 100 µg/ml BSA, and 2 mM ATP or UTP for 45 min at 37°C. Two µl of 100 or 200 nM HARP was added, incubated for 30 min at 30°C and stopped by the addition of 2 µl of 30% (v/v) glycerol-3% (w/v) SDS for analysis on a 5% polyacrylamide gel containing 1xTBE+0.5% SDS. When cited in the main text, the disruption of replication bubbles was quantified as for replication forks, above.

#### Disruption of replication forks containing a poly rG:dC hybrid

Briefly, this fork contains a 29 bp T7 RNA polymerase promoter region in the stem portion followed by a run of 30 dGMP residues in the fork region starting at the start site of transcription in the non-transcribed strand. The oligonucleotides used for construction of this fork are specified in Table S1. It was expedient to synthesize the poly dG-containing top strand by primer extension with 5′-^32^P-labeled primer T7promGG annealed to Bot 5′Δ with exo^-^ Klenow DNA polymerase followed by purification of the 60 nt labeled product on a denaturing gel. Similarly, the template for T7 RNA polymerase transcription was synthesized by primer extension with primer T7promGG annealed to Bot. The yield of RNA from this template was low and not quantifiable and may reflect the need for non-standard transcription conditions and the inability of T7 RNA polymerase to displace the poly rG hybrid, generating lower yields in subsequent rounds (*c.f.*, [28]). The poly rG30 product was 5′-^32^P-labeled with guanylyltransferase. Poly rG30 was annealed to 5′-^32^P-labeled Bot and unlabeled dC30 was annealed to 5′-^32^P-labeled Top. Annealing of the two halves, subsequent incubation with HARP and gel analysis followed the procedures used for the replication bubbles, above.

## Supporting Information

Figure S1 **Proteins purified for this study.** The migration of the size markers for each SDS-PAGE analysis is indicated at the left in kDa.
(TIF)

Figure S2 **The annealing helicase activity of** ***dm*** **HARP expressed in insect cells and in** ***E. coli*** **.** The presence of 150 nM HARP, RPA, topoisomerase I and ATP or UTP are indicated above the gel image. The baculovirus-expressed *dm*HARP displayed lower annealing helicase activity. The asterisk at the left indicates a band that is not normally seen in this plasmid preparation. Lanes with twice the concentration of HARP were removed for this figure.
(TIF)

Figure S3 **HARP facilitates trans annealing of fork and Holliday junction halves.** HARP replication fork regression and Holliday junction migration assays are shown with the labeled precursor halves, final substrates and branch migration products identified at the sides. Eliminating the preannealing step to form the replication fork (lane 2) and Holliday junction (lane 10) indicates that *hs*HARP and *dm*HARP facilitated the annealing of the two halves as evidenced by the reduction in the labeled halves (lanes 3 and 11, respectively). Doubling the concentration of HARP (1x is 10 nM) did not significantly increase fork regression activity (lanes 5–8). Oligo A60 (in red), common to the Holliday and replication fork junctions, was 5′-^32^P-labeled.
(TIF)

Figure S4 **Mismatches at one replication bubble end does not hinder** ***hs*** **HARP activity.** BS+5′-^32^P-labeled B90 and TS+T90 were annealed separately and then combined for 30 min at 37°C, followed by the addition of *hs*HARP (as indicated) for 15 min in the presence of ATP. A 5 bp mismatch at both ends of the replication bubble in the top strand (TS) prevented branch migration (compare lanes 4 and 5 with lanes 2 and 3). Placing a 5 bp mismatch at either end had little (lanes 8 and 9) or no (lanes 6 and 7) effect on branch migration activity. In this assay, the annealing of the two halves was incomplete. Addition of *hs*HARP facilitated annealing as evidenced by the loss of residual, labeled B90:BS half and production of the B90:T90 duplex.
(TIF)

Figure S5 ***hs*** **HARP regression activity on a 3-strand fork junction.** *hs*HARP can disrupt 4-strand (compare lanes 5 and 6) but not 3-strand (compare lanes 2 and 3) fork structures containing a highly stable RNA:DNA hybrid. *dm*HARP was inactive with both structures (lanes 7–10). The hybrid fork drawing at the top defines the shorthand code used for the DNA and RNA strands. Poly dC30 and poly rG30 were separately annealed to the fork ends of the Top and Bottom 60mer strands, respectively, annealed together, followed by the addition of HARP for 15 min at 30°C in the presence of ATP or UTP, as indicated above the gel image. Markers for partial substrates and final product are shown at the left. All nucleic acid components were labeled with the exception of dC30. The dC30:rG30 hybrid product likely co-migrates with rG30 which was in excess in this assay.
(TIF)

Table S1 **Unpublished oligonucleotides used in this study.**
(DOC)
