# Supplementary figures and images for: The Annealing Helicase and Branch Migration Activities of Drosophila HARP
Source: PLoS One. 2014 May 27;9(5):e98173. doi: 10.1371/journal.pone.0098173 (PMC4035279; doi:10.1371/journal.pone.0098173)

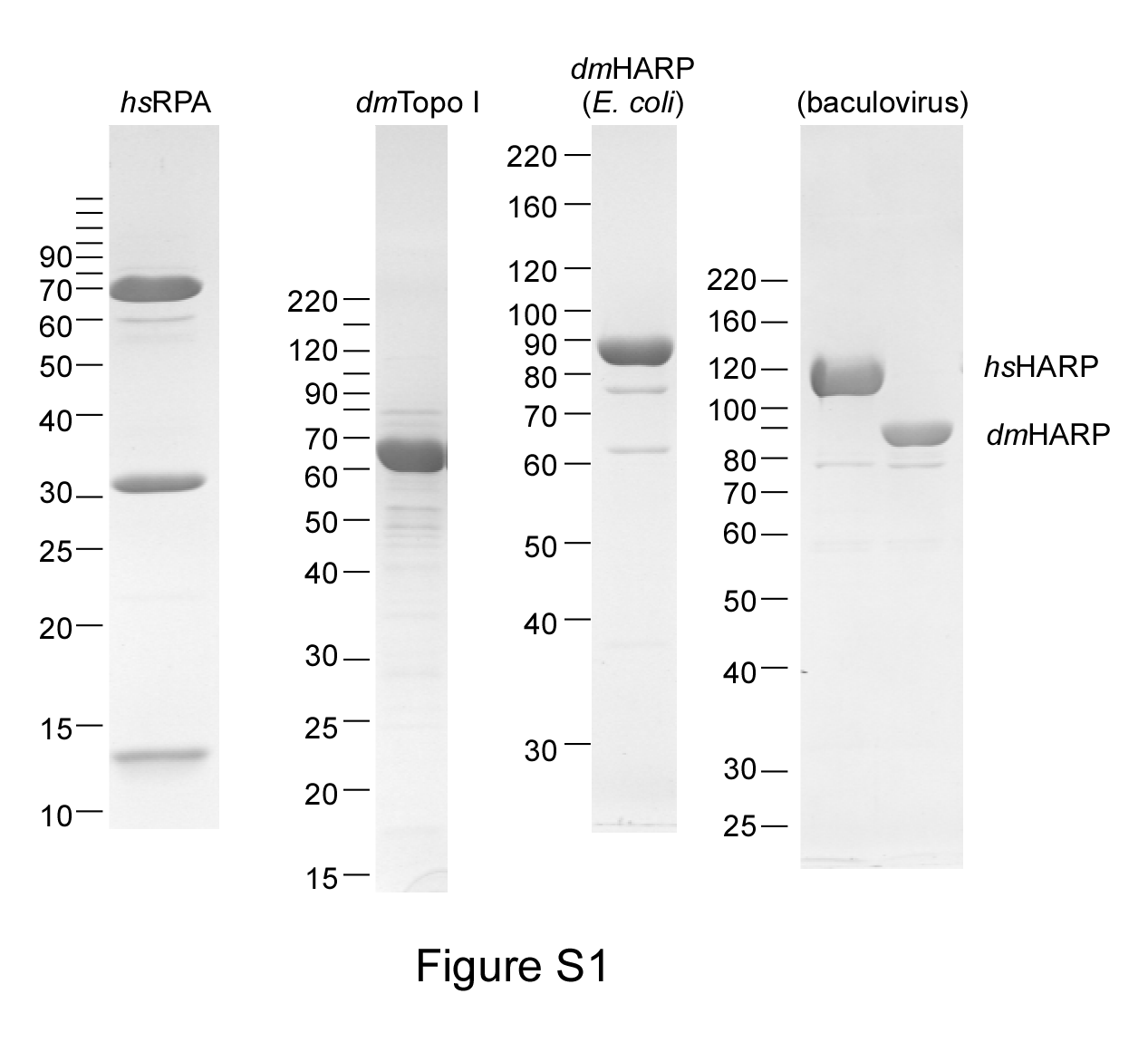

Supplement: Figure S1 — Proteins purified for this study. The migration of the size markers for each SDS-PAGE analysis is indicated at the left in kDa. (TIF) [file pone.0098173.s001.tif]

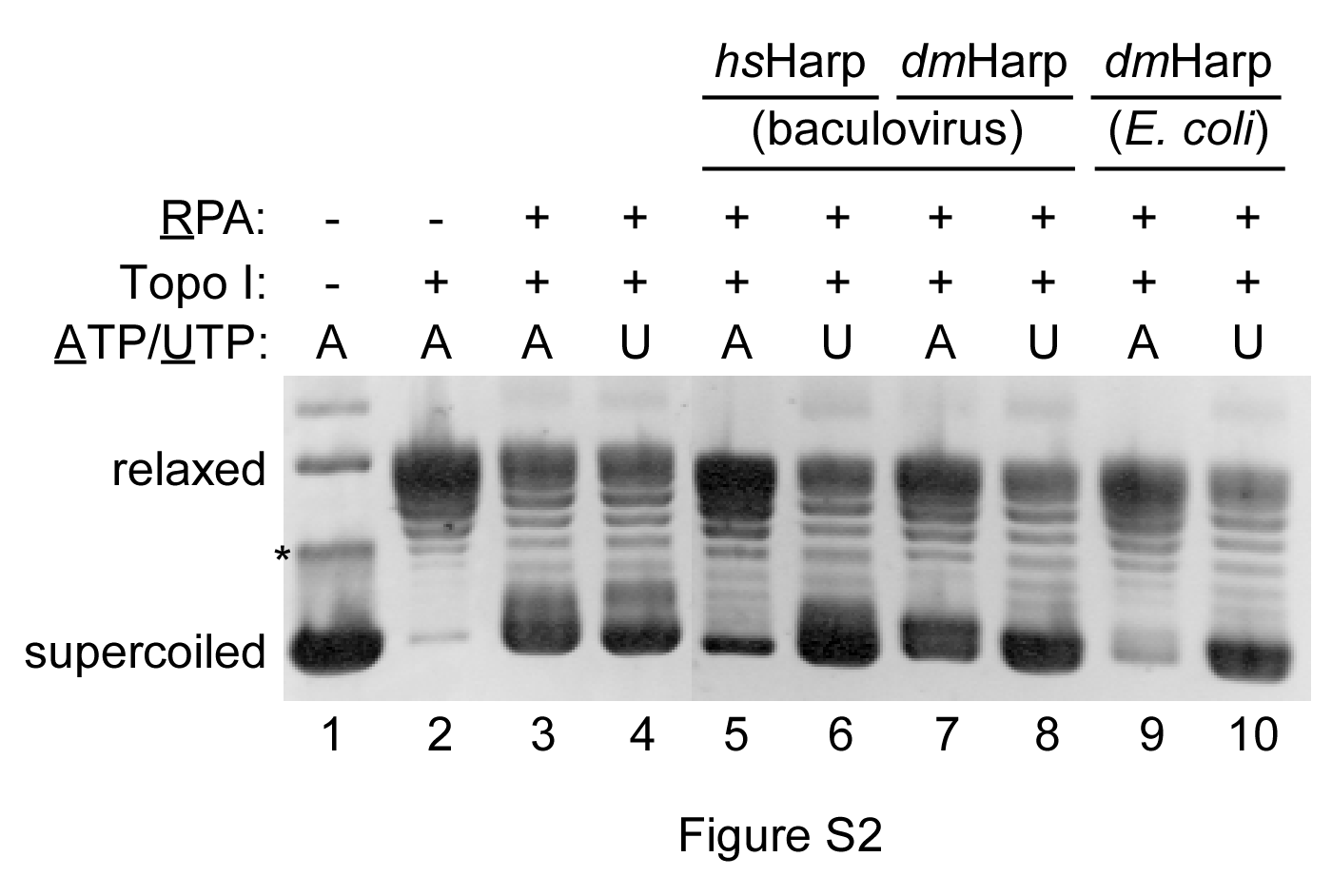

Supplement: Figure S2 — The annealing helicase activity of dm HARP expressed in insect cells and in E. coli . The presence of 150 nM HARP, RPA, topoisomerase I and ATP or UTP are indicated above the gel image. The baculovirus-expressed dmHARP displayed lower annealing helicase activity. The asterisk at the left indicates a band that is not normally seen in this plasmid preparation. Lanes with twice the concentration of HARP were removed for this figure. (TIF) [file pone.0098173.s002.tif]

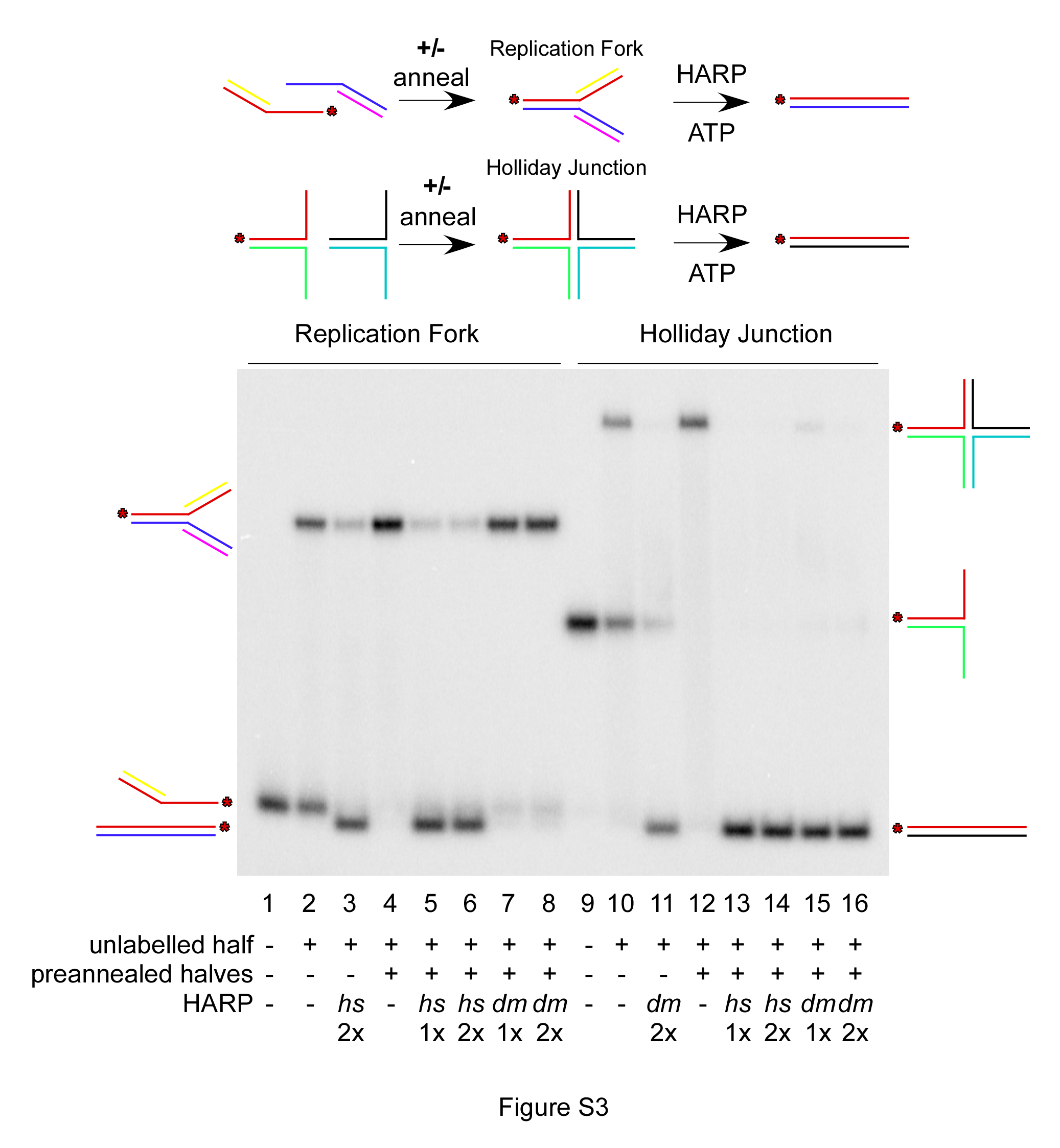

Supplement: Figure S3 — HARP facilitates trans annealing of fork and Holliday junction halves. HARP replication fork regression and Holliday junction migration assays are shown with the labeled precursor halves, final substrates and branch migration products identified at the sides. Eliminating the preannealing step to form the replication fork (lane 2) and Holliday junction (lane 10) indicates that hsHARP and dmHARP facilitated the annealing of the two halves as evidenced by the reduction in the labeled halves (lanes 3 and 11, respectively). Doubling the concentration of HARP (1x is 10 nM) did not significantly increase fork regression activity (lanes 5–8). Oligo A60 (in red), common to the Holliday and replication fork junctions, was 5′-32P-labeled. (TIF) [file pone.0098173.s003.tif]

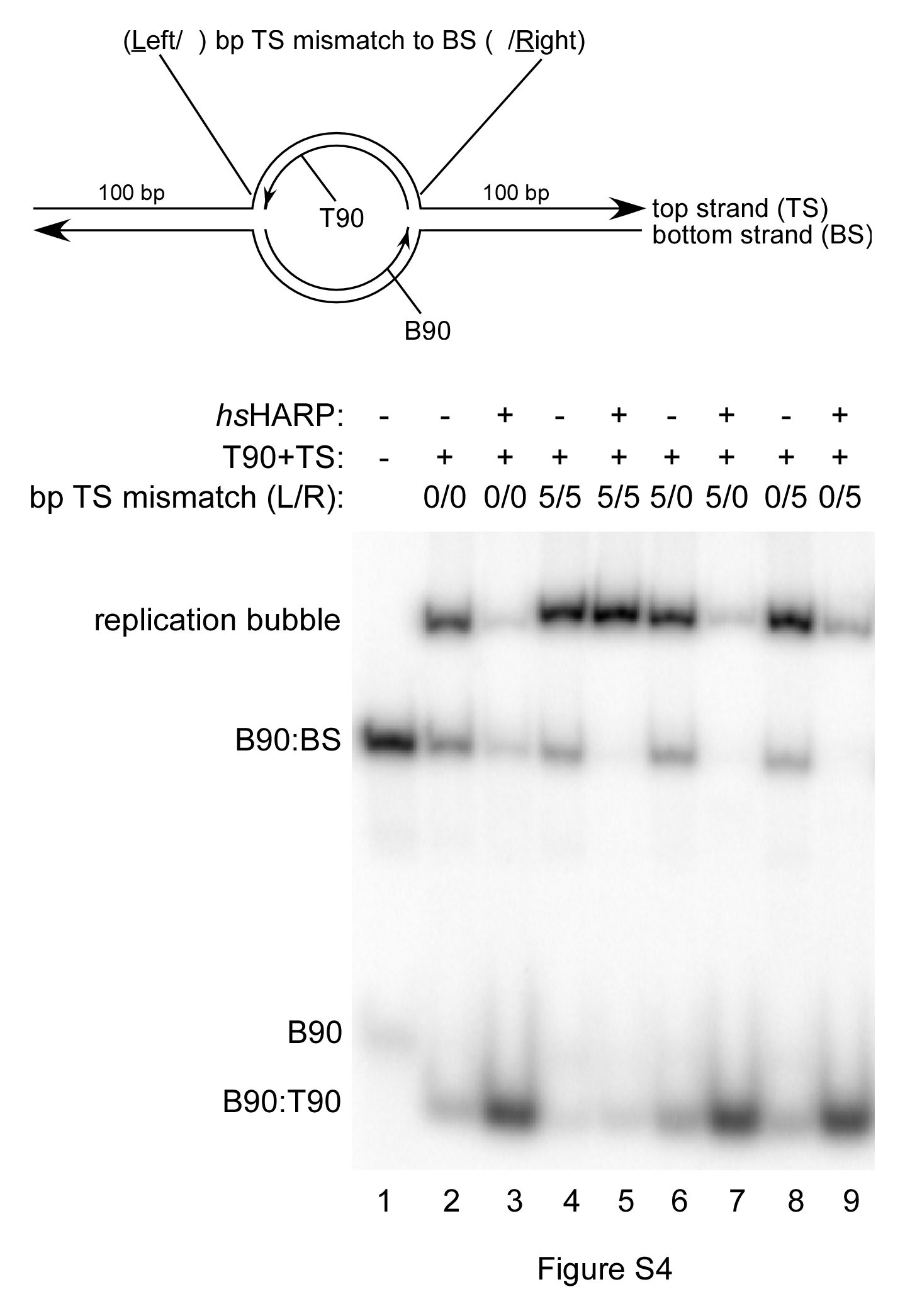

Supplement: Figure S4 — Mismatches at one replication bubble end does not hinder hs HARP activity. BS+5′-32P-labeled B90 and TS+T90 were annealed separately and then combined for 30 min at 37°C, followed by the addition of hsHARP (as indicated) for 15 min in the presence of ATP. A 5 bp mismatch at both ends of the replication bubble in the top strand (TS) prevented branch migration (compare lanes 4 and 5 with lanes 2 and 3). Placing a 5 bp mismatch at either end had little (lanes 8 and 9) or no (lanes 6 and 7) effect on branch migration activity. In this assay, the annealing of the two halves was incomplete. Addition of hsHARP facilitated annealing as evidenced by the loss of residual, labeled B90:BS half and production of the B90:T90 duplex. (TIF) [file pone.0098173.s004.tif]

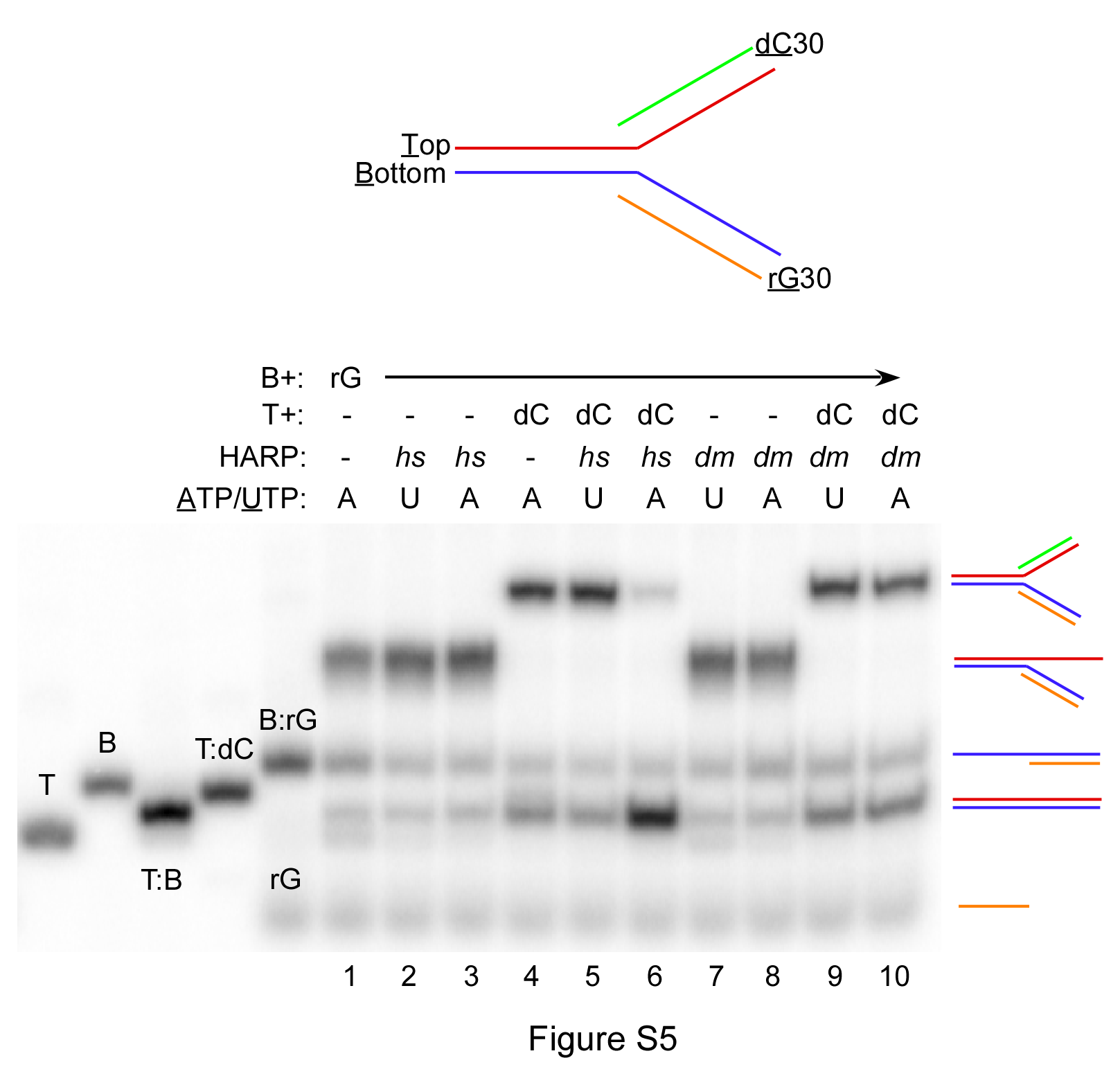

Supplement: Figure S5 — hs HARP regression activity on a 3-strand fork junction. hsHARP can disrupt 4-strand (compare lanes 5 and 6) but not 3-strand (compare lanes 2 and 3) fork structures containing a highly stable RNA:DNA hybrid. dmHARP was inactive with both structures (lanes 7–10). The hybrid fork drawing at the top defines the shorthand code used for the DNA and RNA strands. Poly dC30 and poly rG30 were separately annealed to the fork ends of the Top and Bottom 60mer strands, respectively, annealed together, followed by the addition of HARP for 15 min at 30°C in the presence of ATP or UTP, as indicated above the gel image. Markers for partial substrates and final product are shown at the left. All nucleic acid components were labeled with the exception of dC30. The dC30:rG30 hybrid product likely co-migrates with rG30 which was in excess in this assay. (TIF) [file pone.0098173.s005.tif]
