# Supplementary material for: The Annealing Helicase and Branch Migration Activities of Drosophila HARP
Source: PLoS One. 2014 May 27;9(5):e98173. doi: 10.1371/journal.pone.0098173 (PMC4035279; doi:10.1371/journal.pone.0098173)
Supplement: Table S1 — Unpublished oligonucleotides used in this study. (DOC) [file pone.0098173.s006.doc]

Table S1.

| Name | Sequence (5’ → 3’) |
| --- | --- |
| DL90 | CCGAACACATTCCACTATTTTCGGCTACTATATATATATGTTTTTTTCGCAACTATGTGTTCGCGAAGTAACCCTTCGTGGACATTTGGT |
| B90 | CATCTACAACTCGTGCGACGCATCATTCCTGTGTGAAATTGTTATCCGCTAATCAACCGCAGGCCAAATCCCCATTGAATCTCTACCGAT |
| T90 | ATCGGTAGAGATTCAATGGGGATTTGGCCTGCGGTTGATTAGCGGATAACAATTTCACACAGGAATGATGCGTCGCACGAGTTGTAGATG |
| +150 3’-ribo | TTTACGAGAGAGATGATAGGGTCTGCTTCAGTAAGCCAGATGCTACACArA |
| -100U | CGCCCAATACGCAAACCGCCTCT |
| -150 3’-ribo | GCAGCCGAACGACCGAGCGCAGCGAGTCAGTGAGCGAGGAAGCGGAAGArG |
| +100D | TTAGGCTTGTACATATTGTCGTTAGAACGCGGCT |
| T7PromGG | TGCGACCTGCGGTAATACGACTCACTATAGG |
| Bot 5’Δ | TCCCCCCCCCCCCCCCCCCCCCCCCCCCCCCTATAGTGAGTCGTATTA |
| Bot | TCCCCCCCCCCCCCCCCCCCCCCCCCCCCCCTATAGTGAGTCGTATTACCGCAGGTCGCA |
| dC30 | TCCCCCCCCCCCCCCCCCCCCCCCCCCCCCC |
